# Physical medicine and rehabilitation clinics as transitional care hubs after stroke

**DOI:** 10.3389/fmed.2026.1983310

**Published:** 2026-10-27

**Authors:** John M. Baratta, Elondra Harr, Katherine Cardoza, Mary Jackson, Janet P. Bettger

**Affiliations:** Department of Physical Medicine & Rehabilitation, University of North Carolina at Chapel Hill, Chapel Hill, NC, United States

**Keywords:** bridge clinic, care coordination, physical medicine and rehabilitation, post-discharge follow-up, primary care linkage, social drivers of health, stroke, transitional care

## Abstract

The transition from hospital-based care to community living is a vulnerable period for stroke survivors and caregivers. During this period, patients often experience evolving medical, functional, psychosocial, and social needs while navigating fragmented systems of care. Although guidelines emphasize timely post-discharge follow-up, coordinated discharge planning, rehabilitation continuity, and linkage to primary care and community resources, there remains no consistently implemented model for delivering transitional care after stroke. Outpatient Physical Medicine and Rehabilitation (PM&R) clinics are well positioned to address this gap because physiatrists routinely manage post-stroke disability, functional recovery, secondary complications, therapy coordination, equipment needs, caregiver concerns, and barriers to community reintegration. This Perspective proposes a PM&R-based bridge clinic model for selected stroke survivors discharged home from acute care or inpatient rehabilitation settings, particularly those without established primary care or with barriers to timely outpatient follow-up. In this model, a small interdisciplinary core team, including a rehabilitation physician, advanced practice provider, and social worker or care coordinator, provides time-limited post-discharge follow-up focused on medical, mental health, and functional stabilization, medication reconciliation, rehabilitation planning, durable medical equipment needs, social drivers of health, caregiver support, and primary care linkage. By embedding transitional care within existing outpatient rehabilitation infrastructure, PM&R-based bridge clinics may offer a practical and scalable approach to strengthening continuity across the post-stroke care continuum.

## 1 Introduction

In the United States, stroke is a leading cause of death and disability. Approximately 795,000 people experience a stroke each year, and nearly one in four stroke survivors will have another stroke during their lifetime (1). After discharge, stroke survivors and caregivers often face substantial challenges identifying, accessing, and coordinating the resources needed to support recovery and adjustment to post-stroke life. These challenges may be amplified for individuals with unmet Social Drivers of Health (SDOH) barriers, including financial strain, housing instability, and transportation limitations (2).

The post-stroke period, particularly the shift from hospital-based care to community living, is a critical phase in recovery. Frequently stroke survivors move across multiple care settings, including inpatient care offered in acute hospital units, inpatient rehabilitation facilities, and skilled nursing facilities, and home- and community-based care provided by home health services and outpatient therapy. During this hospital-to-home transition, patients leave high-intensity environments with 24-hour medical oversight and enter community settings with far fewer supports. This complex movement across settings can also contribute to discontinuity of services and gaps in care coordination leading to unmet medical, functional, and psychosocial needs (3).

Common post-discharge challenges include difficulty managing medications and follow-up appointments, limited access to rehabilitation services, and post-stroke complications such as depression, cognitive impairments, and falls (4). Early follow-up after stroke can address these issues and has been associated with lower readmission risk, with guidelines recommending follow-up within the first several weeks after discharge (5). Although stroke survivorship is often managed in primary care, timely post-discharge appointments are not always available, particularly for new patient referrals. Follow-up with neurologists with stroke expertise and outpatient clinic availability is similarly challenging. While waiting for follow up, survivors may experience unresolved medical or functional issues and limited coordination of or access to ongoing care.

Outpatient Physical Medicine and Rehabilitation (PM&R) clinics are well positioned to address these gaps. Physiatrists routinely manage the medical, functional, cognitive, behavioral, and rehabilitation-related consequences of stroke and frequently coordinate with an interdisciplinary team and broader multidisciplinary network including primary care providers, specialists, rehabilitation therapists, durable medical equipment vendors, and community-based services. As a result, PM&R clinics and physiatrist-led outpatient teams may serve as time-limited transitional hubs that help stabilize medical and functional issues, clarify rehabilitation plans, support caregiver needs, and strengthen continuity between inpatient care, outpatient care, and community-based health and social services.

The purpose of this paper is to describe a structured transitional care role for outpatient PM&R clinics in the period immediately following hospital discharge after stroke. We propose a conceptual model in which PM&R clinics function as transitional care hubs to address early post-discharge needs, improve coordination across care settings, and support stroke survivors and caregivers during the vulnerable transition from hospital to community living.

## 2 Transitional care models in medicine

Transitions of care have emerged as an important time period for improving continuity across health care settings, particularly for patients moving from inpatient to outpatient settings. Although transitional care programs vary, commonly described components include discharge or transition planning, early post-discharge follow-up, medication management or reconciliation, patient and caregiver education, information transfer, and communication and coordination between inpatient and outpatient clinicians (6, 7).

Transitional care clinics have been developed in some health systems to provide short-term follow-up for recently discharged patients, particularly those who face barriers to timely primary care access. These bridge clinic models typically offer early post-discharge evaluation, address unresolved medical issues, support medication management, and facilitate connection to longitudinal primary care (8). Many transitional care clinics make use of clinical social workers, care managers, or transitions navigators in conjunction with medical providers to assess behavioral health needs, screen for SDOH concerns, and connect with community resources with the goal of reducing unmet care needs (9). Most described models, however, have been anchored within general medicine or hospital-based programs rather than specialty rehabilitation practices to address more specific needs. This distinction is important for stroke survivors, whose post-discharge needs often span medical stability, functional recovery, caregiver support, and coordination of rehabilitation services.

In stroke populations, reviews of transitional care models demonstrate substantial heterogeneity in intervention design, personnel, and outcomes, highlighting the absence of a standardized approach after hospital or rehabilitation discharge (10–12). Existing guidelines reinforce the need for timely post-discharge follow-up, coordinated discharge planning, rehabilitation continuity, and linkage to primary care and community resources, yet they do not define a standardized model for delivering transitional care after stroke (4, 13). Interventions commonly included patient and caregiver education, follow-up care, medication management, information transfer, and care coordination, but varied in structure and intensity. More recently, the Comprehensive Post-Acute Stroke Services (COMPASS) pragmatic trial evaluated a comprehensive transitional care intervention for patients discharged home after stroke or transient ischemic attack (14). The intervention, which offered individualized care planning and post-discharge follow-up, did not significantly improve functional outcomes or 90-day mortality compared with usual care; however, only 35% of eligible intervention patients attended the COMPASS clinic visit. These findings suggest that transitional care after stroke depends not only on the design of the intervention, but also on whether it can be feasibly embedded into real-world workflows in ways that are acceptable to patients, practical for health systems, and sustainable over time.

Taken together, the existing literature indicates that transitional care remains a critical yet incompletely addressed challenge after stroke. Effective models must be evidence-informed, feasible to implement, and responsive to the medical, functional, and psychosocial needs of stroke survivors during the early post-discharge period.

## 3 The role of physical medicine & rehabilitation clinicians in post-stroke transitional care

One potentially underdeveloped but clinically logical approach for post-stroke transitional care is follow-up in an outpatient clinic led by PM&R. PM&R clinicians (physiatrists) occupy a distinctive role within stroke care that positions them to facilitate transitions across care settings, often working across both inpatient and outpatient environments (13). Physiatrists bring a longitudinal understanding of post-stroke disability trajectories and are well positioned to coordinate therapy services, monitor functional progress, and address common secondary complications, such as mobility limitations, cognitive-communication impairments, spasticity, and pain. In addition, they often continue aspects of medical management after discharge, including reinforcement of secondary stroke prevention strategies, while addressing caregiver needs, equipment concerns, and environmental barriers to recovery. By using the outpatient PM&R clinic as the organizing structure, this model pairs individualized post-stroke assessment and support with an existing clinical infrastructure that can standardize handoffs, coordinate services, and reduce transition-related gaps at the system level. This combination of functional expertise, longitudinal engagement, and interdisciplinary coordination distinguishes PM&R from other specialties and positions rehabilitation clinics as natural hubs for post-stroke transitional care.

Despite this inherent continuity, the transitional role of outpatient PM&R clinics has not been well formalized within stroke systems of care (15, 16). In current practice, rehabilitation follow-up visits are often centered on specific functional concerns or secondary complications rather than structured coordination of broader medical transitions. However, the existing infrastructure of rehabilitation practices, combined with their interdisciplinary orientation and ongoing relationship with stroke survivors after discharge, makes them well suited to support more formalized transitional care activities.

## 4 A proposed PM&R-based bridge clinic model after stroke

### 4.1 Purpose of the bridge clinic

Building on the clinical role of PM&R in post-stroke recovery, this model uses outpatient rehabilitation clinics as bridge clinics for selected stroke survivors following discharge from inpatient care. The PM&R clinic functions as a time-limited transitional care hub during the post-discharge period, providing structured short-term follow-up to stabilize medical and functional issues, coordinate interdisciplinary care planning, and ultimately transition patients to longitudinal outpatient care, including primary care and rehabilitation follow-up as appropriate.

A feasible core team may include a rehabilitation physician, an advanced practice provider, and a social worker or care coordinator. This staffing structure supports medical and functional reassessment, resource linkage, and primary care transition planning while allowing the model to be adapted to local clinic resources. Although post-stroke care often involves multiple disciplines, the bridge clinic model is intended to function as an interdisciplinary approach when implemented through coordinated assessment, shared transition goals, collaborative care planning, and bidirectional communication among the core clinical team and partnering services. This distinction is important because the model depends not only on access to multiple services, but also on structured coordination across those services during the early post-discharge period.

### 4.2 Target population

The proposed model initially focuses on stroke survivors discharged home from acute care or inpatient rehabilitation settings who do not have an established primary care provider or who face barriers to timely outpatient follow-up. This population is particularly high risk for gaps in care, delayed management of vascular risk factors, unmet rehabilitation needs, and SDOH-related barriers.

### 4.3 Proposed workflow

#### 4.3.1 Pre-discharge identification and referral

The bridge clinic process begins before discharge from the hospital or inpatient rehabilitation setting. Prior to discharge, patients are screened to confirm whether they have an established primary care provider and timely follow-up plan, and to identify medical complexity, unmet social needs, and other barriers that may affect outpatient care. Key considerations may include absence of an established primary care provider, anticipated delays in outpatient follow-up, significant functional impairment, unmet social needs, transportation or access-to-care barriers, and whether adequate caregiver support is available to meet the patient's post-discharge needs. Patients meeting locally defined eligibility criteria are referred to the PM&R bridge clinic at the time of discharge, with efforts made to schedule the initial visit before leaving the facility whenever possible.

#### 4.3.2 Early post-discharge bridge visit

An initial bridge clinic visit is ideally conducted within approximately two weeks of discharge, consistent with AHA/ASA guidance recommending a first post-stroke visit within 1–3 weeks after discharge from the hospital or rehabilitation setting (4). This visit serves as a comprehensive reassessment during a period of ongoing recovery and adaptation.

Core components of the visit include medication reconciliation, with particular attention to secondary stroke prevention measures such as blood pressure control, antithrombotic therapy, lipid management, and tobacco cessation. Functional recovery is assessed, including mobility, communication, and activities of daily living, along with evaluation of access to prescribed therapy services and durable medical equipment needed to support independence at home.

Screening for common post-stroke complications, including depression, cognitive impairment, pain, fatigue, and sleep disturbances, is incorporated into the visit. Additional attention is given to equipment needs, home safety, care partner needs, and behavioral health concerns. SDOH-related barriers, including transportation limitations, difficulty accessing care, and financial strain, are also assessed because these factors may significantly affect recovery and follow-up.

The bridge visit may be conducted in person or by telehealth depending on the patient's needs, access barriers, and clinical stability. Telehealth may be well suited for medication reconciliation, review of secondary prevention plans, assessment of therapy access, follow-up on durable medical equipment, screening for mood, cognition, pain, fatigue, sleep disturbance, caregiver burden, and unmet social needs. When video is available, clinicians may also observe elements of mobility, transfers, home setup, and caregiver support. In-person evaluation should be prioritized when patients require a detailed neurologic or musculoskeletal examination, tone or spasticity assessment, equipment fitting, procedures, vital sign assessment, or direct evaluation of safety concerns.

#### 4.3.3 Primary care linkage and transition of care

In addition to addressing stroke-specific rehabilitation needs, a central function of the PM&R bridge clinic is facilitation of primary care linkage for longer-term management. For patients without an established provider, the clinic initiates referral to primary care and may additionally assist with scheduling. For patients with an existing provider but delayed access, the bridge clinic may serve as interim medical support until follow-up can be established.

Where feasible, this process may be strengthened through bidirectional referral pathways to clinical and community resources, allowing providers to receive confirmation of referral receipt, scheduling status, and ongoing care needs. Such closed-loop communication may improve continuity and reduce the likelihood that patients are lost during transition.

Once a primary care relationship is established, a structured “warm handoff” is performed. This may include direct communication with the receiving provider, transfer of relevant clinical information, and clear delineation of ongoing care responsibilities. At that point, longitudinal medical management transitions to primary care, with PM&R continuing to participate in care as indicated for rehabilitation-specific needs.

## 5 Discussion

Stroke survivors frequently leave the hospital or inpatient rehabilitation setting with needs that extend beyond any single specialty visit. In the early post-discharge period, patients and caregivers must manage new medications, therapy referrals, equipment needs, transportation barriers, functional limitations, and evolving neurologic symptoms. Although primary care is essential to long-term stroke survivorship, timely access is not always available. This Perspective proposes that outpatient PM&R clinics can help address this gap by functioning as structured, time-limited bridge clinics for selected stroke survivors.

A PM&R-based bridge clinic model builds on the existing strengths of rehabilitation medicine. Physiatrists routinely evaluate function, disability, therapy needs, care partner needs, equipment, home safety, and secondary complications. These domains are especially relevant in the weeks after discharge, when impairments are changing and outpatient care plans may be in flux. Unlike transitional care models that focus primarily on medical stabilization, a rehabilitation-centered model can integrate medical follow-up with functional recovery and community reintegration. This interdisciplinary orientation distinguishes the bridge clinic from referral-based models in which multiple disciplines may be involved but are not necessarily organized around shared goals or coordinated transition planning.

The proposed model is not intended to replace primary care, neurology, cardiology, or longitudinal rehabilitation care. Rather, it serves as an interim structure during a vulnerable period for higher risk patients. For patients without an established primary care provider, or for those facing delays in outpatient follow-up, the PM&R bridge clinic may provide early reassessment, reinforce secondary prevention plans, identify emerging complications, and facilitate linkage to longitudinal medical care. For patients with existing primary care access, the clinic may still add value by clarifying rehabilitation needs, addressing functional barriers, and communicating stroke-specific concerns to the broader outpatient team.

Successful implementation requires careful patient selection. Not all stroke survivors require a specialty bridge clinic after discharge. A targeted approach may be most feasible, particularly during early implementation. Priority populations may include patients discharged home without an established primary care provider, those with delayed follow-up access, individuals with high medical or social complexity, patients with significant functional impairment, and those with limited caregiver availability or capacity, or transportation barriers. These groups are more likely to experience care gaps and may benefit from structured transitional support. The PM&R-based bridge clinic is logistically feasible but should be evaluated prospectively for patient, family, clinician, and health-system outcomes.

Equity is an important consideration in this model. Stroke survivors with unmet social needs may face disproportionate barriers to follow-up, medication access, transportation, therapy participation, and caregiver support. A structured PM&R bridge visit creates an opportunity to identify SDOH that directly affect recovery, including transportation insecurity, lack of durable medical equipment, limited caregiver availability, financial strain, and difficulty accessing outpatient therapy. While the PM&R clinic cannot resolve all social barriers, it can help identify them early, connect patients with resources, and communicate these needs to others in the care team.

Several limitations should be acknowledged. PM&R clinics vary in staffing, capacity, and integration with inpatient rehabilitation services. Some systems may have embedded care coordinators, social workers, and therapists, while others may rely primarily on physician visits. Outpatient PM&R access may also be limited, and adding bridge visits could worsen clinic capacity if not implemented thoughtfully. Academic systems and public entities may also introduce unique resources and challenges.

In summary, the transition from hospital to home after stroke remains a high-risk and frequently under-structured phase of care. PM&R clinicians are well positioned to address this gap because of their expertise in disability, function, interdisciplinary coordination, and longitudinal recovery. A PM&R-based bridge clinic offers a reasonable and practical model for supporting selected stroke survivors during the early post-discharge period by stabilizing medical and functional needs while assisting the transition to primary care, thereby strengthening the post-stroke care continuum.

## Data Availability

The original contributions presented in the study are included in the article/Supplementary Material, further inquiries can be directed to the corresponding author.
